# Recreational boating degrades vegetation important for fish recruitment

**DOI:** 10.1007/s13280-018-1088-x

**Published:** 2018-08-30

**Authors:** Joakim P. Hansen, Göran Sundblad, Ulf Bergström, Åsa N. Austin, Serena Donadi, Britas Klemens Eriksson, Johan S. Eklöf

**Affiliations:** 1 Baltic Sea Centre, Stockholm University, 106 91 Stockholm, Sweden; 2 AquaBiota Water Research, Löjtnantsgatan 25, 115 50 Stockholm, Sweden; 3 Department of Aquatic Resources, Institute of Coastal Research, Swedish University of Agricultural Sciences (SLU), Skolgatan 6, 742 42 Öregrund, Sweden; 4 Department of Ecology, Environment and Plant Sciences (DEEP), Stockholm University, 106 91 Stockholm, Sweden; 5 Groningen Institute for Evolutionary Life-Sciences (GELIFES), University of Groningen, Nijenborgh 7, 9747 AG Groningen, The Netherlands; 6 Present Address: Department of Aquatic Resources, Institute of Freshwater Research, Swedish University of Agricultural Sciences (SLU), Stångholmsvägen 2, 178 93 Drottningholm, Sweden

**Keywords:** Baltic Sea, Fish reproduction, Lagoons, Macrophytes, Mooring, Shoreline development

## Abstract

Recreational boating increases globally and associated moorings are often placed in vegetated habitats important for fish recruitment. Meanwhile, assessments of the effects of boating on vegetation, and potential effects on associated fish assemblages are rare. Here, we analysed (i) the effect of small-boat marinas on vegetation structure, and (ii) juvenile fish abundance in relation to vegetation cover in shallow wave-sheltered coastal inlets. We found marinas to have lower vegetation cover and height, and a different species composition, compared to control inlets. This effect became stronger with increasing berth density. Moreover, there was a clear positive relationship between vegetation cover and fish abundance. We conclude that recreational boating and related moorings are associated with reduced cover of aquatic vegetation constituting important habitats for juvenile fish. We therefore recommend that coastal constructions and associated boating should be allocated to more disturbance tolerant environments (e.g. naturally wave-exposed shores), thereby minimizing negative environmental impacts.

**Electronic supplementary material:**

The online version of this article (10.1007/s13280-018-1088-x) contains supplementary material, which is available to authorized users.

## Introduction

Aquatic habitats are under increasing pressure from multiple anthropogenic stressors. Along with global warming, eutrophication and fishing, shoreline development is a major factor causing habitat degradation and biodiversity loss in both coastal and freshwater areas (Dudgeon et al. 2006; Halpern et al. 2008). In several European regions, coastal development affects > 80% of the coastline, contributing to degradation and loss of key habitats (Airoldi and Beck 2007). For organisms that are highly dependent on specific habitats during some part of their life-cycle, habitat degradation can have negative effects at the population level (Mumby et al. 2004; Levin and Stunz 2005).

Large-scale disturbances such as eutrophication and fishing have wide-spread and strong effects on coastal ecosystems (e.g. Eriksson et al. 2011). However, also small-scale disturbances, such as those from recreational boat traffic and shoreline construction may have extensive and long-term effects due to cumulative impacts in space and time (Jordan et al. 2009; Eriander et al. 2017), and due to slow recovery of some benthic organisms (Marbà et al. 2003; Forrester et al. 2015). Globally, the number and size of recreational boats have increased with economic growth (e.g. Burgin and Hardiman 2011; Eurostat 2016), and the highest number of boats per capita are found in North America and northern Europe (ICOMIA 2016). This development has led to an increase in boating infrastructure, like jetties and other types of mooring facilities (Campbell and Baird 2009). At least in the Baltic Sea, a disproportionally large part of this development has taken place in shallow, wave-protected areas that also constitute important habitats for benthic vegetation and fish recruitment (Sundblad and Bergström 2014).

Boating can reduce both the abundance and structural complexity of benthic foundation species such as aquatic vegetation (Eriksson et al. 2004; Sandström et al. 2005; Ostendorp et al. 2009) and reef-building corals (e.g. Forrester et al. 2015). These effects can occur through multiple, potentially interacting, mechanisms. First, all types of boats can physically damage benthic organisms through groundings (e.g. Rogers and Beets 2001). Second, the propellers of motor boats can scar, cut or break erect structures like plant shoots or coral colonies (Dawes et al. 1997; Mosisch and Arthington 1998). Third, increased water turbulence from the propulsion system, the boat movement itself, or the wake produced by the boat movement, can increase local hydrodynamic energy thus damaging sensitive organisms, and/or increasing water turbidity by stirring up sediment and therefore cause shading and/or sediment smothering (Mosisch and Arthington 1998; Asplund and Cook 1999). The resuspension of sediments can increase nutrient loadings, locally increase phytoplankton biomass and thus further reduce light penetration (Mosisch and Arthington 1998). Fourth, mooring facilities can directly impact benthic habitats through physical destruction at initiation (Milazzo et al. 2004; Forrester et al. 2015), by shading (Campbell and Baird 2009; Eriander et al. 2017), altered hydrology (Dugan et al. 2011), or continued physical destruction during use (Ostendorp et al. 2009). Finally, boating activities may increase eutrophication due to inadequate waste water treatment, and contribute to chemical pollution through the use of fuel and lubricants in combustion engines, and anti-fouling components on submerged surfaces (Mosisch and Arthington 1998; Burgin and Hardiman 2011).

In areas with particularly intense boating, such as in recreational boat marinas, all the above-mentioned mechanisms could occur simultaneously, with potential additive or synergistic effects. Accordingly, several previous ‘impact versus control’ field surveys indicate negative effects of marinas on the cover and density of aquatic vegetation. Still, the number of studies on the potential environmental effects of recreational boat marinas is low and the existing ones have primarily examined one or two impacted sites (e.g. Marbà et al. 2003; Mueller 2004; Fernández-Torquemada et al. 2005; but see Eriksson et al. 2004). In addition, very few studies have investigated effects of boating activities on the species composition of aquatic vegetation. Such analyses are vital for understanding mechanisms of environmental change, since effects may be species- and/or trait-specific. For example, Eriksson et al. (2004) found lower cover of several rooted angiosperm and characean algae species, but higher cover of a non-attached angiosperm and an attached hard-bottom fucoid algae, in recreational boat marinas and inlets adjacent to ferry routes compared to control sites. This compositional shift may be caused by a higher tolerance to low light conditions and bottom disturbance by species with non-attached free-living growth form, and as a result of erosion of soft substrate by wake and currents exposing hard substrate suitable for attached algae. Since the species composition and morphological traits of aquatic vegetation influence various community- and ecosystem-level properties (e.g. shelter for associated organisms and stabilization of the seabed), changes in such community-level attributes may alter ecosystem structure and function.

Vegetated benthic habitats provide a number of important ecosystem functions. These include sediment stabilization and enhanced water clarity, as well as carbon and nutrient storage, where long-lived rooted vegetation with low decomposition rates seems to be especially important (Madsen et al. 2001; Wang et al. 2016). Aquatic vegetation also provides habitat for numerous other organisms, including fish. The presence and composition of aquatic vegetation have been identified as key factors for coastal fish recruitment, together with abiotic factors such as wave exposure, depth, salinity and temperature (Lazzari and Stone 2006; Snickars et al. 2009, 2010). Species forming large and structurally complex habitats are vital for the recruitment of many coastal fish species (Snickars et al. 2010; Seitz et al. 2014). Accordingly, Sandström et al. (2005) suggested that there were indirect effects of boating on fish recruitment through alterations of vegetation cover and height in the Stockholm Archipelago, in the western Baltic Sea. Currently, there is a need for large-scale assessment of how vegetation abundance and community traits relate to coastal fish production, and to quantify the impact of human activities on habitat-forming vegetation and ecosystem functioning, in order to provide scientific advice for management.

Here, we first examined the effect of recreational boat marinas on the cover, height and composition of aquatic vegetation using a survey in seven marinas paired with physically similar control inlets (open to enclosed bays). Second, we examined the importance of the same vegetation community characteristics for fish recruitment, measured as young-of-the-year (YOY) abundance, using a large-scale dataset of field surveys conducted in inlets (bays, lagoons, sounds and small estuaries) along the entire Swedish east coast (i.e. most of the western Baltic Sea). For the effect of marinas, we hypothesized that (i) the cover and height of aquatic vegetation is lower in marinas than in control inlets due to one or several of the mechanisms described earlier, (ii) the magnitude of these effects increases with increasing development (density of berths), and (iii) the species composition in the vegetation community differs between marinas and controls, particularly regarding rooted soft-bottom species, including sensitive characeans used as environmental indicators (cf. Appelgren and Mattila 2005; Hansen and Snickars 2014). For the analysis of vegetation–fish relationships, we hypothesized that (iv) the abundance of YOY littoral fish species increases with increasing cover of aquatic vegetation in general, but particularly with increasing cover of rooted angiosperms and characean algae, as these increase habitat volume and quality (cf. Sandström et al. 2005; Snickars et al. 2009; Hansen and Snickars 2014).

## Materials and methods

### Effects of marinas on aquatic vegetation

#### Survey design

**Fig. 1:**
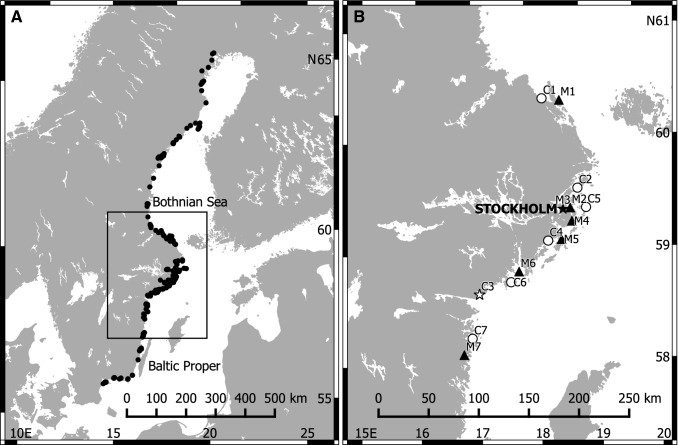
Map of the Baltic Sea showing sampled inlets for the two datasets analysed; **a** inlets used for analysing fish–vegetation relationships, and **b** inlets used for analysing effects of boating activities on vegetation. Letters and numbers in panel **b** refer to pairs (*1*–*7*) of marinas (*M* filled symbols) and control areas (*C* open symbols). The marina/control-pair which was located far apart is indicated by stars

To study the effects of recreational boat marinas on aquatic vegetation, a field survey was conducted in seven marinas and seven paired control inlets in the central part of the non-tidal, brackish Baltic Sea (Fig. 1); an area where recreational boats are common, and kept mainly at berths in the water (Lagerqvist and Andersson 2016). Marinas were defined as shallow inlets that had been allocated for permanent mooring of small (mainly ≤ 12 m) motorboats and sailboats for personal transport and/or recreational use during the boating season (late spring to early autumn). Marinas were chosen to form a pressure gradient, from small boat harbours with few berths to extensive marinas with a high number of berths (Table 1; method for estimation of number of jetties and berths is described in Appendix S1). The resulting gradient in boating pressure (13–391 berths inlet^−1^, corresponding to 3–46 berths ha^−1^) and morphometry of the inlets (shallow enclosed to deeper open bays) represent the range of those for recreational boat marinas in shallow inlets in the area. The number of berths of the examined inlets has been approximately constant over the last decade (Fig. S1).

**Table 1:** Abiotic variables of the examined marinas and control areas. See Appendix S1 for method description

Variables	Category	Pairs														*t*/*F*-values^a^	*p* values
		1		2		3		4		5		6		7			
		$\bar{x}$	± SE	$\bar{x}$	± SE	$\bar{x}$	± SE	$\bar{x}$	± SE	$\bar{x}$	± SE	$\bar{x}$	± SE	$\bar{x}$	± SE		
Berths (no.)	Marina	13		26		391		160		39		45		25			
	Control	1		1		9		3		2		0		0			
Jetties (no.)	Marina	6		6		11		13		7		6		4			
	Control	1		0		3		1		1		0		0			
Water surface area (ha)	Marina	4.38		5.66		8.52		9.74		2.07		3.40		3.02		0.47	0.655
	Control	8.15		1.24		5.57		10.2		3.38		1.75		3.13			
Water retention time (days)	Marina	8.0		1.4		0.08		0.06		15.7		1.5		1.8		− 1.30	0.240
	Control	15.1		0.4		0.02		0.11		25.6		1.6		1.0			
Wave exposure (Log_10_ m^2^ s^−1^)	Marina	3.1		3.4		3.3		3.4		3.2		4.6		3.2		− 1.41	0.209
	Control	3.6		3.5		3.9		3.6		2.9		4.7		3.0			
Maximum depth (m)	Marina	3		6		20		6		3		3		3		1.00	0.356
	Control	3		6		15		6		3		3		3			
Sampling depth (m)	Marina	1.5	± 0.2	1.9	± 0.2	1.5	± 0.3	1.8	± 0.1	1.3	± 0.2	1.4	± 0.2	1.2	± 0.2	6.09	0.016
	Control	1.7	± 0.1	2.6	± 0.2	1.7	± 0.3	1.8	± 0.2	2.2	± 0.3	1.4	± 0.2	1.5	± 0.1		
Salinity (PSU)	Marina	6.6	± < 0.1	4.8	± < 0.1	4.8	± < 0.1	5.4	± < 0.1	5.2	± < 0.1	6.1	± < 0.1	5.5	± < 0.1	1.68	0.242
	Control	6.6	± < 0.1	5.2	± < 0.1	6.4	± < 0.1	5.7	± < 0.1	5.0	± < 0.1	6.4	± < 0.1	5.3	± < 0.1		
Total nitrogen conc. (µmol L^−1^)	Marina	23.3	± 0.9	20.7	± 1.6	20.5	± 1.0	19.9	± 0.4	63.5	± 1.8	21.3	± 1.3	32.2	± 2.1	1.14	0.326
	Control	21.8	± 0.8	20.5	± 0.5	23.6	± 0.4	19.1	± 0.7	33.2	± 0.8	22.7	± 1.9	27.7	± 0.9		
Total phosphorous conc. (µmol L^−1^)	Marina	0.90	± 0.11	0.51	± 0.05	0.59	± 0.06	0.42	± 0.02	3.48	± 0.07	1.04	± 0.09	1.73	± 0.14	1.03^b^	0.350
	Control	0.90	± 0.05	0.49	± 0.01	0.98	± 0.01	0.54	± 0.02	1.07	± 0.02	0.80	± 0.06	0.57	± 0.02		
Turbidity (NTU)	Marina	2.7	± 0.3	6.5	± 0.1	4.9	± 0.1	7.7	± 0.1	5.3	± 0.1	2.0	± 0.1	2.8	± 0.3	0.61^b^	0.464
	Control	3.4	± 0.2	2.6	± 0.3	1.8	± 0.1	1.7	± 0.1	2.6	± 0.6	2.5	± 0.5	6.5	± 0.1		
Dominant substrate (> 90%)	Marina	M, ST, B, BR		M, G		M, SA		M, SA		M		M, SA, G		M		0.25	0.784
	Control	M		M, G		M, SA, ST		M, SA, G, ST		M, BR		M, SA, ST		M			

*M* mud, *SA* sand, *G* gravel, *ST* stones, *B* boulders, *BR* bedrock

^a^Differences between marina and control sites in area, retention time, wave exposure and maximum depth were tested with paired *t*-tests (inlets as replicates), while differences in sampling depth, salinity, nutrient concentrations and turbidity were tested with mixed effects models (stations as replicates), where the random factor inlet was nested within marina–control pairs. Differences in substrate composition were tested with a PERMANOVA with marina–control pairs as random strata

^b^Tests were performed on log_10_-transformed values

Since aquatic vegetation in Baltic Sea inlets is influenced by a number of abiotic factors, such as openness towards the sea, wave exposure and depth (e.g. Appelgren and Mattila 2005), each marina was paired with a control area with as similar morphometry as possible, but without (or with very few) mooring facilities. Control areas were located adjacent (13–40 km) to each marina, except one which was located further south (Fig. 1). Initial analysis showed no significant difference in the morphometric variables, nor in the measured abiotic variables between marinas and controls (*p* ≥ 0.2, Table 1), except for depth (see Statistical analyses).

#### Field sampling

Sampling of aquatic vegetation was done in late summer (August to early September) 2014, when the vegetation reaches its maximum cover and biomass. Within each inlet, 6–8 stations (higher number with increasing inlet area) were randomly positioned at 0.5–3 m depth (i.e. within the dominant depth interval) and > 30 m apart. The stations consisted of a 5-m radius circle (ca. 80 m^2^), within which the per cent cover of aquatic vegetation was visually estimated by a free-diver. First, the total area covered by vegetation (0–100%, hereafter ‘total vegetation cover’) was estimated. Second, the percentage cover of each taxon was estimated separately and identified to species in the field, except filamentous algae which were later identified in the laboratory (Table S1). The filamentous algae grew mainly free-lying or as loosely attached epiphytes. Similarly, coarsely structured algae (e.g. *Fucus vesiculosus*) grew both free-lying and attached to the available hard substrate in the otherwise soft-bottom dominated inlets. The cumulative sum of all taxa, and that of rooted angiosperms and charophytes, was calculated for each station (hereafter ‘cumulative’ and ‘rooted’ vegetation cover). Cumulative and rooted vegetation cover could exceed 100% when taxa grew on top and/or overlay each other. The water depth (nearest 0.1 m) and canopy height of the vegetation (nearest 0.05 m) was measured at five random points within each circle and averaged before statistical analyses. Canopy height was defined as the maximum vegetation height above the seabed, excluding the tallest 10% of vegetation in a 0.5 × 0.5 m quadrat.

#### Statistical analyses

All analyses were performed in R version 3.3.2 (R Core Team 2016). Differences in vegetation cover and height between marinas and control inlets were analysed with mixed effects models (Table S2). Marina/control was included as a fixed factor, while inlets nested in marina–control-pairs were included as random factors. As sampling depth was somewhat shallower in marinas than in controls (*p* < 0.02; Table 1), depth was initially tested but was removed because it did not contribute to the model (*p* > 0.12). Normally distributed residuals were achieved by arcsine transformation of total vegetation cover, square-root transformation of rooted vegetation cover, and logarithmic transformation of vegetation height (log_10_). Estimates of *p*-values were based on Satterthwaite’s approximation for denominator degrees of freedom and pseudo-*R*^2^ was separated into variance explained by only the fixed factors (marginal *R*^2^) versus the fixed and random factors together (conditional *R*^2^; Table S2).

The relationship between magnitude in boating pressure and effects on aquatic vegetation was examined by linear regression (normal error distribution), using density of berths (log_10_-transformed number of berths per water surface area of the marina inlets) as explanatory factor for the difference in vegetation cover between marinas and controls (average cover in marina subtracted by average cover in the paired control inlet).

Differences in vegetation composition were examined by a permutated analysis of variance (PERMANOVA), using Bray–Curtis dissimilarity calculations (Table S2), with marina–control-pairs as strata. Depth was initially tested, but was non-significant (*p* = 0.13) and removed from the final model. Prior to analysis, single-sample occurrence of three species was removed. Differences in species abundance were examined using the *SIMPER* routine (Table S2).

### Influence of aquatic vegetation on coastal fish

#### Data description

The influence of aquatic vegetation on coastal fish production was assessed using 3132 stations from 200 inlets (bays, lagoons, sounds and small estuaries) across the entire Swedish east coast, covering a substantial range of climatic and hydrographic variables represented by gradients from south to north and inner to outer parts of the archipelagos (Fig. 1a). The database of juvenile fish abundance has been collated from a large number of different sources, mainly from national and local surveys and monitoring programmes, as well as research projects, and consists in total of over 16 000 stations collected between 1978 and 2016 (Swedish University of Agricultural Sciences). In this study, only surveys using comparable fish and vegetation sampling methods were selected. These methods included the use of a free-diver, percent estimates of vegetation cover and a similar sampling unit (5-m radius) during mid to late summer (late July to mid-September). Only inlets with a minimum of five stations were included (maximum = 64, dependent on inlet area). The YOY fish assemblages have been surveyed using small underwater detonations (non-electric system with 10-g primers), yielding a quantitative sample of fish (≤ 20 cm) (Snickars et al. 2007). All floating fishes were netted and sunken fishes were collected by a free-diver. The fishes were identified to species and counted. The free-diver also assessed habitat characteristics, including per cent cover of individual vegetation species, using the same method as described earlier. Estimates of ‘total vegetation cover’ and vegetation height are, however, missing in these surveys. Based on the comparable methods, the data covered 9 years (2007–2015) and of the 200 inlets, 27 had been sampled during 2 years and 25 during 3–6 years (yielding a total of 316 bay–year combinations). By using such a large dataset, the analyses are robust to the large inter-annual variability in YOY fish abundance (Kallasvuo et al. 2016). However, the data primarily reflects the relationship between vegetation and fish in undisturbed areas, as highly impacted areas generally have been avoided, making it unsuitable to assess direct shoreline development effects (e.g. berth density) on vegetation and YOY fish abundance. Juvenile fish was sampled also in the seven marina/control-pairs, but given the normally large variation in fish recruitment, 14 inlets were considered inadequate to estimate how fish abundance depends on both vegetation and boating activities. Consequently, although lower pike (*Esox lucius*) abundance has previously been observed in marinas (Sandström et al. 2005) we could not find a significant difference in fish abundance between marinas and control inlets in our smaller dataset (*p* > 0.1).

Species included in the YOY fish assemblage have a preference for high temperature and a moderate or strong dependence on vegetation during spawning and/or an association to vegetation during some early life-stage (Sandström et al. 2005). The YOY fish assemblage included Northern pike (*E. lucius*), Eurasian perch (*Perca fluviatilis*), ruffe (*Gymnocephalus cernuus*), and cyprinids (mainly roach *Rutilus rutilus*) (Table S4). Pike and perch were analysed separately since they as adults are piscivores, providing a suite of ecosystem services, and are of special interest for environmental conservation and fisheries management (e.g. Östman et al. 2016), while the other species stay planktivorous and/or benthivorous. As inter-specific interactions may partly determine the fish assemblage’s spatial distribution, it was judged that analysing the total abundance of the assemblage was most appropriate for detecting vegetation effects.

To account for the influence of two main environmental gradients on fish composition—the (1) inner to outer archipelago zones, and (2) wave-sheltered (enclosed) to exposed (open) morphology of the inlets (Snickars et al. 2009)—all inlets were assigned to one of three inlet types; “inner sheltered”, “outer sheltered” or “outer exposed”. The classification was based on averages per inlet from a model of surface wave exposure (see Appendix S1 for description), and the number of water bodies from the offshore areas (based on the Water Framework Directive typology of the coast), where the two outermost coastal water bodies were considered “outer”, and “inner” if three or more coastal water bodies separated the inlet from the offshore areas. The fourth possible category (“inner exposed”) contained only three inlets and was excluded from further analyses.

#### Statistical analyses

Generalized linear mixed effect models were used to relate fish abundance to vegetation cover (Table S2). Inlet type (three levels) and vegetation cover (continuous variable) were treated as fixed effects (after checking that vegetation cover was not affected by inlet type), while inlet was included as a random effect, as multiple samples were collected within the same inlet. A compound symmetry auto-correlation structure was included to account for repeated measures. For each response variable (number of YOY pike, perch, and the rest of the assemblage), two (separate) models with different vegetation variables were tested; cumulative vegetation cover and rooted vegetation cover (Pearson *ϱ* = 0.68, *n* = 3132, *p* < 0.001). Vegetation variables were normalised in order to make model estimates comparable between response variables. The highly skewed distribution of YOY fish abundance, which contained many zeroes and occasionally very high values, did not adequately fit a Poisson distribution. To handle the overdispersion and reduce bias in parameter estimates, an observation level random effect was added (Table S2). Moreover, the YOY assemblage data were fourth-root transformed to fit assumptions. Models were evaluated using scaled residuals and tested for overdispersion and zero-inflation (Table S2), and pseudo-*R*^2^ was calculated as for vegetation (described earlier).

Since the sampled area stretched > 1100 km in a north–south direction, latitude was included as a predictor in all initial models. Latitude was never significant (*p* > 0.7), except for juvenile pike (*p* < 0.001 for both cumulative cover and rooted vegetation) showing higher juvenile pike abundance at southern latitudes. However, since latitude only marginally affected the vegetation estimates (second decimal), and there was no interaction between latitude and vegetation (*p* > 0.5), we only report the model without latitude to enhance comparison between models. The resulting models were used to predict relative fish density as a function of vegetation depending on inlet type, which reflect the response at the station scale. At the inlet scale, the effect of vegetation on YOY fish abundance was visualised by binning averages per inlet and year at 20% cover intervals. The number of inlets per 20% bin was for cumulative vegetation *n* = 12, 45, 83, 84 and 92, and for rooted vegetation *n* = 41, 97, 98, 54 and 26. Different sizes of bins were tested, but did not influence the result.

## Results

### Effects of marinas on aquatic vegetation

**Fig. 2:**
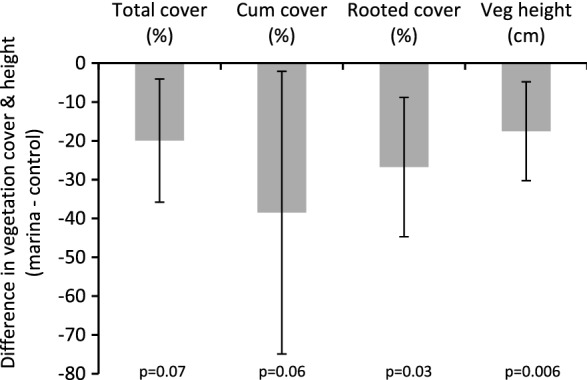
Mean difference (± 95% CI) in total, cumulative and rooted vegetation cover (%), and vegetation height (cm) between marinas and control areas. Significance is given below the bars

There was a clear effect of marinas on vegetation status (Fig. 2). The cover of rooted vegetation was 27% lower in the marinas than in the controls and there were also trends of lower total and cumulative vegetation cover in the marinas (Table 2; Fig. 2). Lowest observed cover in the marinas was 20, 29 and 11% for total, cumulative and rooted vegetation cover, respectively (Table 3). In addition, vegetation canopy height was 0.2 m lower in the marinas than in control inlets (Table 2; Fig. 2).

**Table 2:** Differences in vegetation response variables between marinas and control areas. Estimates show results for fixed effect from general models (Gaussian distribution) with inlets nested in marina/control-pairs as random factors (transformed data, see Method section)

Response variables	Est.	SE	*t*-values	*p*-values	*R*^2^ marginal	*R*^2^ conditional
Total vegetation cover	− 0.22	0.10	− 2.19	0.071	0.08	0.44
Cumulative vegetation cover	− 38.5	18.6	− 2.07	0.060	0.13	0.48
Rooted vegetation cover	− 2.38	0.83	− 2.87	0.029	0.19	0.47
Vegetation height	− 0.19	0.06	− 3.35	0.006	0.13	0.15

**Table 3:** Berth density and mean vegetation cover and height per inlet (± SE) of the examined marinas and control areas

Variables	Category	Pairs													
		1		2		3		4		5		6		7	
		$\bar{x}$	± SE	$\bar{x}$	± SE	$\bar{x}$	± SE	$\bar{x}$	± SE	$\bar{x}$	± SE	$\bar{x}$	± SE	$\bar{x}$	± SE
Berths per water surface area (no. ha^−1^)	Marina	3		5		46		16		19		13		8	
	Control	0.1		0.8		1.6		0.3		0.6		0.0		0.0	
Total vegetation cover (%)	Marina	20	8	88	8	47	6	68	13	29	9	58	11	70	9
	Control	62	12	90	4	93	2	68	8	64	10	79	5	64	10
Cumulative vegetation cover (%)	Marina	48	20	97	10	53	7	113	24	29	9	82	9	111	16
	Control	72	17	120	9	164	13	83	9	107	10	162	11	97	21
Rooted vegetation cover (%)	Marina	45	20	15	2	20	3	16	6	11	6	20	7	43	9
	Control	53	11	8	3	84	11	46	9	58	9	54	8	53	10
Vegetation height (cm)	Marina	32	10	21	4	17	4	25	5	24	8	17	3	17	1
	Control	40	9	37	10	33	6	16	2	73	13	34	12	43	16

**Fig. 3:**
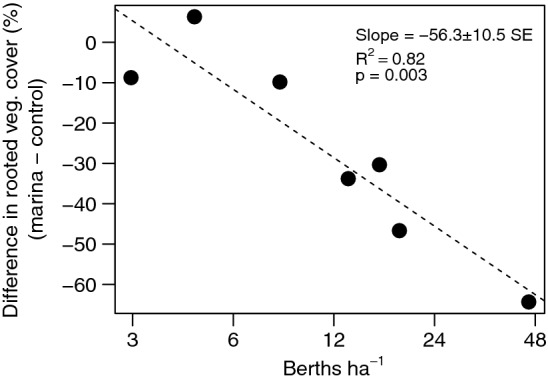
Relationship between berth density in marinas and difference in rooted vegetation cover between marinas and control areas. Note the logarithmic *x*-axis

The strength of the negative effect of marinas on the rooted vegetation cover increased with the density of berths, from almost no effect in marinas with few berths to 30–64% lower cover in the most developed marinas (> 10 berths ha^−1^; *t* = − 5.35, *p* = 0.003; Fig. 3). The other vegetation variables were not affected by density of berths in the marinas (*p* > 0.2).

To facilitate comparisons with other studies we also present degradation effects by recalculating the absolute differences in cover to relative proportions ($\bar{x}$ cover in marinas/$\bar{x}$ cover in controls). The average cover of rooted vegetation in the marinas was 65% of that in control areas, while for both total and cumulative cover it was 73%. In the most developed marinas the cover of rooted vegetation was 19–37% of that in controls.

Species composition also differed between marinas and controls (PERMANOVA, *F* = 4.78, *p* < 0.001). Both common rooted angiosperms (e.g. *Stuckenia pectinata*, *Potamogeton perfoliatus* and *Najas marina*) and a number of rare species, which have been previously identified as sensitive to eutrophication and boat-mediated disturbance (e.g. *Chara aspera* and *Chara tomentosa*; Hansen and Snickars 2014), had lower cover in marinas (Table S3). Also, the cover of filamentous algae was lower in marinas than in controls.

### Influence of aquatic vegetation on coastal fish

**Fig. 4:**
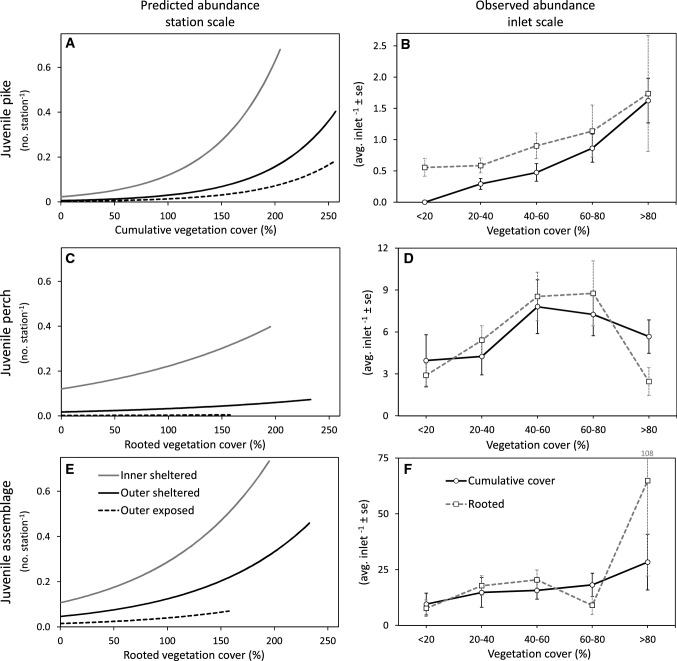
Abundance of juvenile (YOY) fish in relation to vegetation cover, for pike (top row, panels **a** and **b**), perch (mid row, panels **c** and **d**) and the rest of the assemblage which benefit from warm water and vegetation during the earliest live-stages (bottom row, panels **e** and **f**) at two spatial scales. The station scale (left column, panels **a**, **c**, **e**) show model predicted abundance at the station level (model scale) for three inlet types, and the *x*-axes reflect the type of vegetation with strongest effect on YOY abundance (Table 4). Observed abundances visualised at the inlet scale (right column, panels **b**, **d**, **f**) are based on binned abundances at 20% interval for cumulative cover of all vegetation species and rooted species alone for all inlet types combined

The analyses of the large-scale fish survey data showed clear positive relationships between the abundance of YOY pike, perch and the rest of the assemblage to both the cumulative vegetation cover and the rooted vegetation cover. Cumulative cover of all vegetation species had a stronger positive effect on juvenile pike abundance than the cover of rooted vegetation species alone (Table 4). For perch and the juvenile assemblage, rooted vegetation appeared to have a slightly stronger positive effect than cumulative cover, but the standard error of the estimates overlapped (Table 4). All species were more abundant in sheltered inlets in the inner archipelago, than in sheltered and exposed inlets in the outer archipelago, as indicated by the intercepts for inlet type (Fig. 4a, c, e). Inlet type was significant (*p* < 0.01) in all models (data now shown).

**Table 4:** Relationships between cumulative and rooted vegetation cover and abundance of juvenile pike, perch and the warm-water and vegetation associated fish assemblage. Estimates show results for the fixed effects from generalized models (Poisson distribution) with inlets and inlet per year, as well as an observation level, random effects

Predictor variables	Response variables	Est.	SE	*z*-values	*p*-values	*R*^2^ marginal	*R*^2^ conditional
Cumulative vegetation cover	Pike	0.73	0.06	12.82	< 0.001	0.11	0.58
	Perch	0.17	0.09	1.95	0.051	0.12	0.46
	Juvenile assemblage	0.33	0.05	6.37	< 0.001	0.10	0.41
Rooted vegetation cover	Pike	0.33	0.05	6.19	< 0.001	0.07	0.55
	Perch	0.23	0.08	2.72	0.007	0.12	0.46
	Juvenile assemblage	0.37	0.05	7.47	< 0.001	0.10	0.40

By pooling the vegetation in the surveyed inlets into 20% cover categories (bins), the positive effect of vegetation on YOY fish abundance at the inlet scale could be visualised (Fig. 4b, d, f). The abundance of both pike and the assemblage increased strongly with increasing average vegetation cover (both cumulative and rooted) in the inlets. Perch abundance increased with vegetation cover, but decreased above 80% cover.

## Discussion

The results of our field survey suggest that recreational boating negatively influences the structure of aquatic vegetation. Coastal inlets utilized for extensive mooring (i.e. marinas) had lower cover and height, and a different composition of aquatic vegetation, than similar control inlets with no or very few moorings. Furthermore, the extent of habitat degradation appeared to increase with the density of berths in the marinas. These results are in line with the few previous quantitative studies available, which show that vegetation abundance in marinas is about 30–80% of that in control areas (Marbà et al. 2003; Eriksson et al. 2004; Mueller 2004; Fernández-Torquemada et al. 2005). In the present study, the average proportion of vegetation was ca. 70% of that in the paired control inlets, while in the most extensive marinas (> 10 berths ha^−1^) the proportion of rooted vegetation was ca. 20–40% compared to controls. Interestingly, the magnitude of these effects is comparable to that of moderate eutrophication (comparison with Baltic Sea soft-bottom areas with a similar nutrient regime; Wikström et al. 2016). Although the mechanisms behind the observed differences between marinas and control inlets were not examined, the lower cover of rooted vegetation in marinas and the near absence of species known to be outcompeted at low light conditions (e.g. *C. aspera*, Blindow and Schütte 2007, and *C. tomentosa*, Appelgren and Mattila 2005), indicate that disturbance of sediments and resuspension of particles may be an important mechanism. Our one-time measurements of turbidity showed no differences between marinas and control areas, but since these measurements are only a snapshot in time and turbidity interacts with vegetation and varies over the season and even shorter periods (e.g. Madsen et al. 2001), this potential mechanism remains to be studied in more detail. However, the lower vegetation height in marinas suggests that other boat-mediated mechanisms are also important, since poor light conditions often incite elongation in many vegetation species (Barko and Smart 1981; Blindow and Schütte 2007). A likely explanation is the physical disturbance by boats, for example propeller scarring and damaging currents (Dawes et al. 1997; Asplund and Cook 1999). Finally, the lower cover of filamentous algae in the marinas points towards a third possible mechanism; non- or loosely-attached species could be washed ashore or out of the inlets as a result of boat-generated wake and currents (Roos et al. 2003). Here, a contributing factor could be the lower cover of rooted foundation species that act as substrate for these epiphytic algae.

Loss of aquatic vegetation affects various ecosystem functions in the coastal zone (e.g. Orth et al. 2006). The particular lower cover of rooted vegetation in the studied marinas can reduce sediment stabilization, nutrient uptake and storage (e.g. Madsen et al. 2001; Wang et al. 2016). Such ecosystem effects may be non-linear (with thresholds), as suggested for eelgrass (*Zostera marina*) on the Swedish west coast (Moksnes et al. 2018) where areal losses exceeding a certain threshold results in increased sediment resuspension and proliferation of drifting algal mats that through negative feedback-mechanisms prevent recovery of the seagrass meadows. Moreover, the lower cover and height of vegetation in the examined marinas results in a decreasing extent (volume) of vegetation habitat for associated organisms, potentially affecting abundance and productivity.

Here, using an extensive dataset on plant and fish assemblages in 200 coastal inlets, we show that the cover of aquatic vegetation plays an important role for fish production, where the vegetation is utilized as spawning and/or nursery habitat (Snickars et al. 2009, 2010; Hansen and Snickars 2014). The strong positive relationship to vegetation cover shown for pike is consistent with earlier findings (Sandström et al. 2005; Craig 2008). Although cumulative cover had a stronger effect than rooted vegetation, observed pike densities at the inlet scale was higher for rooted vegetation. Interestingly, pike was absent from inlets with a mean cumulative cover < 20% but present if the sparse vegetation consisted of rooted plants, indicating that rooted plant habitats also play a role, particularly at low vegetation density. The lowest cumulative vegetation cover in our studied marinas was just above 20%, suggesting that the fish recruitment habitat was degraded but not absent. For juvenile perch there was a hump-shaped relationship with vegetation cover at the inlet scale. Most likely, intermediate cover of vegetation provides perch juveniles with shelter from predators, whereas high vegetation cover reduces their foraging ability and growth (Diehl and Eklöv 1995). This probably also explains why the effect of vegetation cover was lower for perch compared to pike and the rest of the fish assemblage, since the fitted model estimated a linear effect (Table 4). Even though we were unable to explicitly test the effect of marinas on juvenile fish, due to very few data from heavily developed areas, the two analyses viewed together suggest that recreational boating, by changing the vegetation composition and reducing the cover and height, can reduce the production of juvenile fish. Similar results have been found in surveys of marinas in the past (Sandström et al. 2005), and highlight the importance of potential indirect (cascading) effects of negative impacts on foundation species like aquatic vegetation.

Since the availability of shallow, wave-sheltered and vegetated fish reproduction habitats is limited in the Baltic Sea Archipelago areas (Sundblad et al. 2011; Kallasvuo et al. 2016), and availability has been shown to constrain adult population densities (Sundblad et al. 2014), our results suggests that intense shoreline development and boating could have indirect negative effects on coastal fish populations. Simultaneously, pike and perch can be important for the foundation species in these systems, through the suggested top-down control of filamentous algal growth (Östman et al. 2016), creating a risk for negative feedback loops following losses of predatory fish and/or degraded vegetation in recruitment habitats. Given that the development of marinas has increased over time, and that most of this development has taken place in environments that constitute the optimal habitats for recruitment of many coastal fish species (Sundblad and Bergström 2014), this study pinpoints the need to in greater detail assess the magnitude of impact, and the mechanisms involved, in order to identify sustainable use levels. Until such studies have been conducted, we recommend that as a precautionary approach coastal constructions and associated boating should to the greatest extent be allocated to more disturbance tolerant environments, for example naturally wave exposed shores and non-vegetated deeper areas (Sandström et al. 2005), in order to minimize potential negative effects on important, more sensitive coastal habitats such as shallow wave-sheltered vegetated inlets.

Restrictions on shoreline development and boating activities in sensitive areas do not have to stand in direct conflict with the interests of boat owners, since experience of relatively pristine nature is highly valued and often the motivation for pleasure boat use (Lagerqvist and Andersson 2016). Sport fishing is also of great recreational and economic value in the Baltic Sea region, with pike and perch being among the most important species (SwAM 2014). Also non-fishing citizens value coastal nature, and non-market benefits such as improving the preservation of currently pristine areas, habitat forming vegetation and large predatory fish stocks are high (Kosenius and Ollikainen 2015). Maintaining well-functioning coastal ecosystems that balances the use and protection of vegetated habitats will ensure a sustained delivery of ecosystem services and benefits for human well-being.

## Conclusion

We conclude that recreational boating and related moorings are associated with altered species composition and reduced cover and height of aquatic vegetation that constitute important habitats for juvenile fish. We recommend that as a precautionary approach, mooring constructions and associated boating should as far as possible be allocated to more disturbance tolerant environments (wave exposed and/or deeper shorelines) to reduce negative environmental effects.

## Electronic supplementary material

Below is the link to the electronic supplementary material.

Supplementary material 1 (PDF 766 kb)
