# Supplementary material for: Recreational boating degrades vegetation important for fish recruitment
Source: Ambio. 2018 Aug 30;48(6):539–51. doi: 10.1007/s13280-018-1088-x (PMC6486933; doi:10.1007/s13280-018-1088-x)
Supplement: Supplementary file 1 — Supplementary material 1 (PDF 766 kb) [file 13280_2018_1088_MOESM1_ESM.pdf]

Ambio

Electronic Supplementary Material

Title: **Recreational boating degrades vegetation important for fish recruitment**

Authors: Joakim P. Hansen, Göran Sundblad, Ulf Bergström, Åsa N. Austin, Serena Donadi, Britas Klemens Eriksson, Johan S. Eklöf

Appendix S1. Method description for abiotic variables

Number of jetties and berths

The number of jetties and berths were counted in each inlet (i) during the field sampling and (ii) from satellite images provided by Google Earth Pro (Version 7.1.5.1557) and the Swedish mapping, cadastral and land registration authority (Lantmäteriet, Metria) for the year of field sampling (2014). All types of jetties were counted, including a few piers, docks and boat-houses. Similarly, all types of berths were counted, including berths on jetties, piers, docks, boat-houses and permanent mooring buoys, as well as berths where no boats were seen at visit or at the time the satellite image was taken. In cases when the size of an empty berth was uncertain (due to lack of boats or structures clearly defining the size), the average boat size in the marina was used to approximate size of the berth. Consequently, the number of berths is an approximation of the actual boating pressure in the inlets. The number of jetties and berths in the inlets were further examined 6–12 years back in time (depending on available satellite images) and found to be approximately constant (Fig. S1).

Water retention time

Water retention time of the inlets (T_y, days) was calculated as $\mathrm{Ln}(T_y) = -4.33 \times \sqrt{E_a} + 3.49$, where E_a is the topographic openness of an inlet (i.e. degree of isolation from the sea) which was calculated according to $E_a = 100 \times A_t/a$, where A_t is the smallest cross-sectional area of an inlets opening, and a is the water surface area of the inlet (Persson et al. 1994; Håkansson 2008). The cross-sectional area, A_t, was calculated from depth measurements in the field and distance measurements conducted using GIS-methods on satellite images (QGIS v. 2.12.3 (2016) and Google Earth Pro). Water surface area of the inlets, a, was also measured by GIS-methods. Estimates of water retention time based on the above described method correlates well with empirical data of surface water retention time (R^2=0.93; Persson et al. 1994).

Wave exposure

Surface wave exposure ($m^2\ s^{-1}$) was estimated for a central point in each bay using a GIS-based wave model, based on averages of fetch calculations from 16 compass directions with wind conditions over a 5-year period and accounting for diffraction effects (Isæus, 2004; Sundblad et al. 2014a). The two

exposure categories ‘sheltered’ and ‘exposed’ used for analyses of fish abundance was divided at 10 000 m^2s^{-1}, since larvae and juveniles of the studied species are most often found below this value (Sundblad et al. 2009; 2011; 2014b).

Water depth

Maximum water depth of the inlets was obtained from nautical charts prior to field sampling. At field sampling, water depth was measured at five random sites at each station (nearest 0.1 m) and averaged per station.

Salinity, nutrients and turbidity

Water was sampled randomly at each field station at 0.5 m depth before midday. Salinity (practical salinity unit, PSU) was measured directly using a Multi 340i voltmeter (WTW Germany). Since turbidity may be affected by light and temperature, the water samples were stored dark and cold for ca. 6 hours until measuring turbidity in nephelometric turbidity units (NTU) (three estimates per station) using a Aquafluor® turbidimeter (Turner Designs, USA). Water for nutrient analysis were frozen and stored dark, and later analysed for total nitrogen and phosphorus concentrations at Stockholm University’s accredited laboratory (Sweden's national accreditation body, Swedac) through Segmented Flow Analysis (SFA, modified after Hansen and Koroleff 1999) with ALPKEM O I Analytical (Flow Solution IV), after digestion with acid-persulphate at high temperature (modified after Valderrama 1981).

Substrate composition

Composition of substrates at each field station was estimated simultaneously as the vegetation cover survey (by a free-diver). The contribution of seven different substrates of the seabed was estimated as a continuous percentage fraction of a maximum cover of 100%. The substrate types were bedrock, large boulders (> 600 mm), boulders (200–600 mm), stones (20–200 mm), gravel (2–20 mm), sand (0.2–2 mm) and finer sediment (mud).

References

Hansen, H. P., and E. Koroleff. 1999. Determination of nutrients. In *Methods of Seawater Analysis*. 3rd edition, ed K. Grasshoff, K. Kremling and M. Ehrhardt, 159–228. Wiley-VCH

Håkansson, L. 2008. Factors and criteria to quantify coastal area sensitivity/vulnerability to eutrophication: presentation of a sensitivity index based on morphometrical parameters. *International Review of Hydrobiology* 93: 372–388.

Isæus, M. 2004. Factors structuring *Fucus* communities at open and complex coastlines in the Baltic Sea. PhD Thesis. Department of Botany, Stockholm University, Sweden.

Persson, J., L. Håkanson, and P. Pilesjö. 1994. Prediction of surface water turnover time in coastal waters using digital bathymetric information. *Environmetrics* 5: 433–449.

QGIS Development Team. 2016. QGIS Geographic Information System. Open Source Geospatial Foundation Project. http://www.qgis.org/.

Sundblad, G., M. Härmä, A. Lappalainen, L. Urho, and U. Bergström. 2009. Transferability of predictive fish distribution models in two coastal systems. *Estuarine, Coastal and Shelf Science* 83: 90–96.

Sundblad, G., U. Bergström, and A. Sandström. 2011. Ecological coherence of marine protected area networks: A spatial assessment using species distribution models. *Journal of Applied Ecology* 48: 112–120.

Sundblad, G., T. Bekkby, M. Isæus, A. Nikolopoulos, K.M. Norderhaug, and E. Rinde. 2014a. Comparing the ecological relevance of four wave exposure models. *Estuarine, Coastal and Shelf Science* 140: 7–13.

Sundblad, G., U. Bergström, A. Sandström, and P. Eklöv. 2014b. Nursery habitat availability limits adult stock sizes of predatory coastal fish. *ICES Journal of Marine Science* 71: 672–680.

Valderrama, J.C. 1981. The simultaneous analysis of total nitrogen and total phosphorus in natural waters. *Marine Chemistry* 10: 109–122.

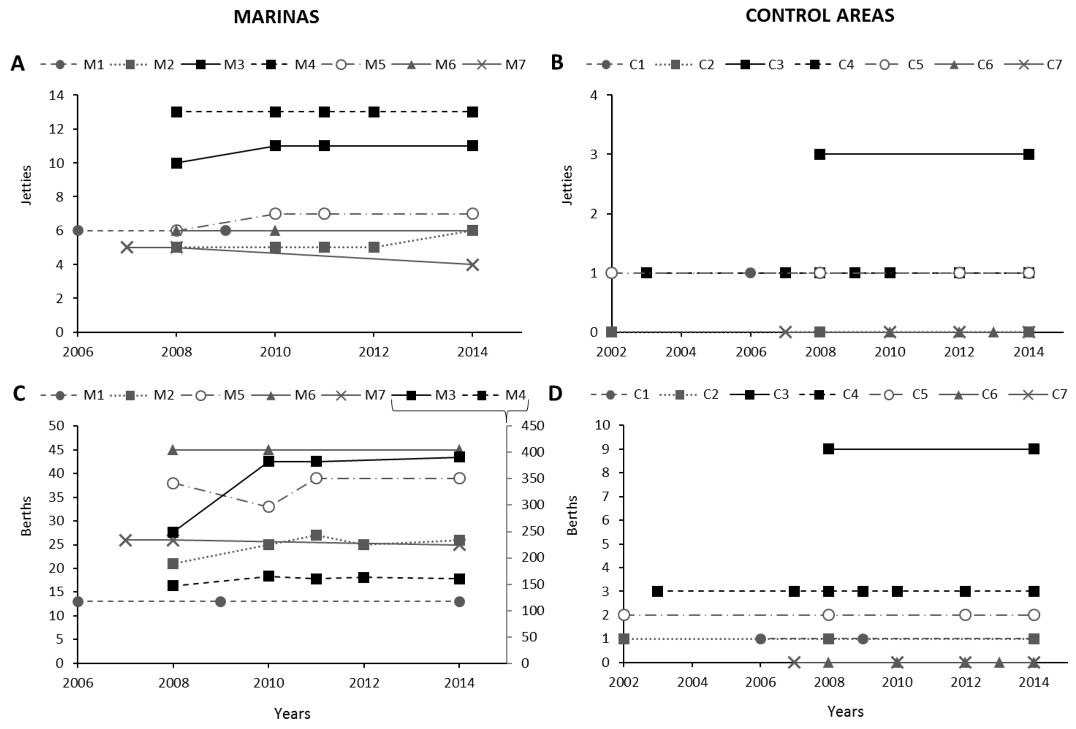

Figure S1. Development of jetties (upper row, panels **a** and **b**) and berths (lower row, panels **c** and **d**) over years in the studied marinas (left column, panels **a** and **c**) and control areas (right column, panels **b** and **d**), until the field-sampling year 2014. Note that number of berths for marina *M3* and *M4* are given on right axis in panel **c**.

Table S1. Taxa of 'filamentous algae' recorded in the studied inlets, sorted after frequency of occurrence in the analysed samples. Taxonomic names follow the Swedish Taxonomic Database (www.dyntaxa.se)

**Taxa**	**Phylum/Superphylum**	**# Samples**
Cladophora glomerata	Chlorophyta	20
Lyngbya	Cyanobacteria	10
*Ulva**	Chlorophyta	9
Cladophora fracta	Chlorophyta	8
Rhizoclonium	Chlorophyta	8
Unidentified uniseriate Chlorophyta, Charophyta or Cyanobacteria		7
Cladophora rupestris	Chlorophyta	4
Rivularia atra	Cyanobacteria	4
Aegagropila linnaei	Chlorophyta	3
Ceramium tenuicorne	Rhodophyta	2
Mougeotia	Charophyta	2
Pylaiella littoralis	Heterokonta	2
Ectocarpus siliculosus	Heterokonta	2
Chaetomorpha linum	Chlorophyta	1
Dictyosiphon foeniculaceus	Heterokonta	1
Rhodochorton purpureum	Rhodophyta	1
Spirogyra	Charophyta	1
Zygnema	Charophyta	1
Oedogonium	Chlorophyta	1

*Fine tubular spp. Mainly *U. clathrata* vel *flexuosa* and *U. procera* vel *prolifera*

Table S2. Specification of statistical methods and software packages used in the program R (version 3.3.2; R Core Team 2016) for the different response variables, with sample unit and dependent factors listed

Response variables	Sample unit	Fixed dependent factors	Random factors	Statistic tests	R packages
• Total vegetation cover<br>• Cumulative vegetation cover<br>• Rooted vegetation cover<br>• Vegetation height	Station	• Marina/control (2 levels)	• Inlet (marina-control pair)	Mixed effects models	*lme4* (Bates et al. 2015) with *lmer* function and *lmerTest* for *p*-value estimates (Kuznetsova et al. 2016), and *MuMIn* for pseudo-R^2 (Nakagawa and Schielzeth 2013)
Differences in<br>• Total vegetation cover,<br>• Cumulative vegetation cover,<br>• Rooted vegetation cover, and<br>• Vegetation height<br>between marinas and control inlets	Inlet	• Number of berths per water surface area of the marinas (berths ha^{-1}, continuous)		Linear regression	*stats* with *lm* function (R Core Team 2016)
• Vegetation species composition	Station	• Marina/control (2 levels)	• Marina-control pair	PERMANOVA and SIMPER	*Vegan* with *adonis* and *simper* functions (Clarke and Warwick 1994; Oksanen et al. 2016)
• Juvenile pike abundance<br>• Juvenile perch abundance<br>• Juvenile assemblage abundance	Station	• Inlet type (3 levels) and<br>• Cumulative vegetation cover (continuous), or<br>• Rooted vegetation cover (continuous)	• Inlet (year)<br>• Observational random effect (Harrison 2014)	Mixed effects models	*lme4* with *glmer* function and *MuMIn* for pseudo-R^2

Assumptions of statistical distributions were examined using residual plots in the *stats* and *DHARMa* (Hartig 2016) packages

References

Bates, D., M. Mächler, B. Bolker, and S. Walker. 2015. Fitting linear mixed-effects models using lme4. *Journal of Statistical Software* 67: 1–48.

Clarke, K. R., and R. M. Warwick. 1994. *Changes in marine communities: An approach to statistical analysis and interpretation.* Natural environment research council, Plymouth marine laboratory.

Harrison, X.A. 2014. Using observation-level random effects to model overdispersion in count data in ecology and evolution. *PeerJ* 2: e616.

Hartig, F. 2016. DHARMa: Residual diagnostics for hierarchical (multi-level/mixed) regression models. *R package version 0.1.2.* https://CRAN.R-project.org/package=DHARMa.

Kuznetsova, A., P.B. Brockhoff, and R.H.B. Christensen. 2016. lmerTest: tests in linear mixed effects models. *R package version 2.0-33*. https://CRAN.R-project.org/package=lmerTest.

Nakagawa, S., and H. Schielzeth. 2013. A general and simple method for obtaining R2 from generalized linear mixed-effects models. *Methods in Ecology and Evolution* 4: 133–142.

Oksanen, J., F.G. Blanchet, M. Friendly, R. Kindt, P. Legendre, D. McGlinn, P.R. Minchin, R.B. O'Hara, et al. 2016. vegan: community ecology package. *R package version 2.4-1*. http://cran.r-project.org/package=vegan.

R Core Team. 2016. *R: A language and environment for statistical computing*. Vienna: R Foundation for Statistical Computing. http://www.R-project.org/

Table S3. Result of a SIMPER test of differences in species cover between marinas and control inlets. Taxa listed according to taxonomic and functional group (i.e. if rooted) and average contribution to overall dissimilarity. Columns show average ($\bar{x}$) contribution to overall dissimilarity, standard deviation (SD) of contribution, average cover per inlet type, and *p*-value for test of differences between inlet types. Significant differences ($p < 0.05$) are indicated by bold font. Taxonomic names follow the Swedish Taxonomic Database (www.dyntaxa.se)

Taxon/Taxonomic and functional group	$\bar{x}$ overall	SD	$\bar{x}$ marina	$\bar{x}$ control	p-values
**Rooted angiosperms**					
Stuckenia pectinata	**0.10**	**0.11**	**8.39**	**16.48**	**0.004**
Potamogeton perfoliatus	**0.07**	**0.10**	**4.00**	**13.02**	**0.002**
Najas marina	**0.06**	**0.13**	**0.98**	**8.79**	**<0.001**
Myriophyllum spicatum	0.04	0.08	6.22	1.62	1.000
Ruppia cirrhosa	**0.03**	**0.07**	**1.04**	**5.48**	**<0.001**
Myriophyllum sibiricum	0.01	0.04	0.39	1.83	0.184
Ranunculus circinatus	0.01	0.02	0.93	0.55	1.000
Callitriche hermaphroditica	0.01	0.01	0.89	0.024	1.000
Zannichellia palustris	0.003	0.011	0.41	0.12	1.000
Ranunculus peltatus subsp. *baudotii*	**0.001**	**0.003**	**0.022**	**0.190**	**0.040**
**Rooted macroalgae**					
Chara baltica	**0.01**	**0.03**	**0.17**	**1.33**	**0.004**
Chara tomentosa	**0.01**	**0.02**	**0.022**	**0.79**	**<0.001**
Chara aspera	**0.003**	**0.013**	**0.000**	**0.60**	**<0.001**
Chara globularis vel *virgata*	**0.001**	**0.003**	**0.065**	**0.071**	**0.024**
**Non-rooted angiosperm**					
Ceratophyllum demersum	0.03	0.07	2.39	2.43	0.999
**Non-rooted, attached or non-attached macroalgae**					
Filamentous algae	**0.18**	**0.14**	**18.48**	**38.48**	**0.002**
Fucus vesiculosus	0.18	0.17	31.15	21.55	1.000
Chorda filum	0.01	0.02	0.26	0.98	0.159
Monostroma balticum	0.004	0.019	0.87	0.000	1.000
Furcellaria lumbricalis	**0.003**	**0.011**	**0.000**	**0.52**	**<0.001**

Table S4. Taxonomic list of juvenile (young of the year) fish species in the analysed dataset. Taxonomic names follow the Swedish Taxonomic Database (www.dyntaxa.se). Temperature and vegetation preference follow Sandström et al. 2005 and FishBase (www.fishbase.org) and references therein

Latin names of species	Common names	Temperature preferences*	Vegetation preferences**
Perca fluviatilis	eurasian perch	high	moderate
Gymnocephalus cernuus	ruffe	high	moderate
Leuciscus idus	ide	high	moderate
Rutilus rutilus	roach	high	strong
Abramis brama	bream	high	strong
Blicca bjoerkna	white bream	high	strong
Esox lucius	northern pike	high	strong
Tinca tinca	tench	high	strong
Scardinius erythrophthalmus	rudd	high	strong
Carassius carassius	crucian carp	high	strong

* low = optimum temperature for consumption < 20 ºC and optimum temperature for survival of embryos < 10 ºC. high = optimum temperature for consumption > 20 ºC and optimum temperature for survival of embryos > 10 ºC.
** strong = species depending on vegetation as spawning substrate and with both larval and juvenile life stages strongly associated to vegetation, moderate = species depending on vegetation as spawning substrate and/or associated to vegetation during some early life-stage, weak = other spawning substrate and not associated to vegetation during any of the early life stages

Sandström, A., B.K. Eriksson, P. Karås, M. Isæus, and H. Schreiber. 2005. Boating and navigation activities influence the recruitment of fish in a Baltic Sea Archipelago area. *Ambio* 34: 125-130.
